# Response to Training in Emotion Recognition Function for Mild TBI/PTSD Survivors: Pilot Study

**DOI:** 10.3390/brainsci15070728

**Published:** 2025-07-08

**Authors:** J. Kay Waid-Ebbs, Kristen Lewandowski, Yi Zhang, Samantha Graham, Janis J. Daly

**Affiliations:** 1 Department of Veterans Affairs (VA), Rehabilitation Research and Development, Brain Rehabilitation Research Center, Gainesville, FL 32608, USA; yi.zhang@va.gov (Y.Z.); samantha.graham2@va.gov (S.G.); jjd17@case.edu (J.J.D.); 2 Department of Speech, Language, and Hearing Sciences, College of Public Health and Health Professions, University of Florida, Gainesville, FL 32611, USA; kristen.lewandowski@va.gov; 3 Department of Physical Therapy, College of Public Health and Health Professions, University of Florida, Gainesville, FL 32611, USA; 4 Department of Neurology, School of Medicine, Case Western Reserve University, Cleveland, OH 44106, USA

**Keywords:** traumatic brain injury (TBI), post-traumatic stress disorder (PTSD), emotion function, affect recognition, prosody recognition

## Abstract

Background/Objectives: For those with comorbid mild traumatic brain injury/post-traumatic stress disorder (mTBI/PTSD), deficits are common with regard to recognition of emotion expression in others. These deficits can cause isolation and suicidal ideation. For mTBI/PTSD, there is a dearth of information regarding effective treatment. In pilot work, we developed and tested an innovative treatment to improve recognition of both affect (facial expression of emotion) and prosody (spoken expression of emotion). Methods: We enrolled eight Veterans with mTBI/PTSD and administered eight treatment sessions. Measures included the following: Florida Affect Battery (FAB), a test of emotion recognition of facial affect and spoken prosody; Attention Index of the Repeatable Battery for the Assessment of Neuropsychological Status (RBANS); and Emotion Recognition Test (ERT), a speed test of facial emotion recognition. Results: There was a significant treatment response according to the FAB (*p* = 0.01, effect size = 1.2); RBANS attention index (*p* = 0.04, effect size = 0.99); and trending toward significance for the ERT (0.17, effect size 0.75). Participants were able to engage actively in all eight sessions and provided qualitative evidence supporting generalization of the training to interpersonal relationships. Conclusions: Our data show promising clinical potential and warrant future research, given the importance of developing novel interventions to train and restore recognition of emotion in Veterans with mTBI/PTSD.

## 1. Introduction

Normal function of the emotion recognition process is essential for healthy interpersonal relationships. Accurate emotion recognition in everyday life requires encoding and synthesis of different types of incoming stimuli from others, including both static and dynamic facial expressions, as well as prosody (spoken vocal loudness, pitch, and intensity). Deficits in emotion recognition have been associated with poor quality of interpersonal relationships, loss of employment, behavioral problems, reduced social reintegration, social isolation, and even suicide [1,2,3,4]. Emotion recognition deficits are common in mild traumatic brain injury (mTBI) alone [5,6] and in post-traumatic stress disorder (PTSD) alone [6,7,8]. Comorbid mild TBI/PTSD (mTBI/PTSD) results in more severe symptoms, in general, versus either mTBI or PTSD alone [9,10]. Yet, emotion recognition, specifically, has not been well studied in Veterans with comorbid mTBI and PTSD. This is important, given the prevalence of these two comorbidities resulting from the nature of the injuries for Veterans [9,10,11,12,13].

Others have recently highlighted the theoretical advantages of a variety of multimodal trainings in other fields and across an array of other populations [14]. However, to our knowledge, there are no publications, to date, describing an effective treatment that would address the array of possible deficits in emotion recognition for those with co-morbid mTBI/PTSD. Though important prior studies have been conducted, the current state-of-the-art is limited in at least four ways, as follows: (1) no studies of emotion recognition training in mild TBI; rather, only a few existing studies that are focused on moderate/severe TBI [3,15,16,17]; (2) no studies of emotion recognition training for comorbid mTBI/PTSD; rather, existing studies focused on moderate/severe TBI [3,15,16,17,18,19]; (3) for this population, inclusion of only static facial recognition training, that is, no inclusion of training on dynamic facial expressions [18,19]; (4) lack of inclusion of prosody training , auditory stimuli from vocal production; that is, only facial affect training (i.e., training in recognition of emotion through only facial expression) [3,15,16,17].

It is critical to develop and test promising treatment options given the lack of effective interventions, as well as the number of mTBI injuries [20] and Veterans with comorbid mTBI/PTSD [9,10,11,12,13]. These persons are in need of treatment for deficits in recognition of emotion expression through facial affect and prosody. Therefore, the purpose of this pilot was to develop and pilot-test the innovative Multi-Modal Affect Recognition Intervention (MMART). The MMART is constructed using multiple experiential training stimuli, including two visual arrays and one auditory array of stimuli. These multiple modes of training were first presented separately, and then in combination. In this manner, the MMART is targeted to improve recognition of emotion expressed through facial affect and prosody stimuli in Veterans with comorbid mTBI/PTSD.

## 2. Methods

The design of this study was a single-group intervention pilot study, with pre-/post-treatment evaluations. All subjects completed the eight intervention sessions in a clinical laboratory setting, as well as the pre-/post-evaluations, with the exception of two participants with a missing post-treatment assessment in one of the secondary measures, as detailed in the results section.

### 2.1. Participants

This study was a single-group, pre-/post-treatment study, conducted in a clinical research setting, under the oversight of the institutional official IRB Human Subjects Protection Board at the University of Florida #201800127 (date of approval 22 June 2018). Recruitment was conducted by word of mouth, flier, and referral from psychologists, physicians, and other healthcare professionals aware of the study. Written informed consent was obtained from all subjects involved in the study. Inclusion criteria were as follows: Veterans with a diagnosis of mTBI recorded in the medical records by a physician and at least a year prior, based on VA/Department of Defense guidelines; similarly with a diagnosis of PTSD in the medical record; score, at least one standard deviation below the mean on a subscale of the Florida Affect Battery indicating a deficit in emotion recognition; age, 18–55; corrected vision within normal limits; able to hear 25 dB at 500 to 4000 Hz. Exclusion criteria were as follows: history of schizophrenia, bipolar disorder, chronic medical or neurological diseases other than TBI.

### 2.2. Intervention

In this pilot, we tested the Multi-Modal Affect Recognition intervention (MMART) in Veterans with mTBI/PTSD. The MMART, developed in our research program, is a theoretically and experientially based treatment protocol. Treatment is targeted to improve recognition of emotion expressed by others that is communicated through their facial expression and vocal expression. There are innovative aspects inherent in the MMART. First, for example, it employs dynamic facial expressions for training; that is, the user works with not only photographs, but also with videos of facial expressions of different emotions. Second, the MMART includes training not only in recognizing facial expression of emotion, but also importantly includes training in the recognition of vocal expression. Vocal expression of emotion includes loudness, pitch, and intensity, and together, these factors are termed ‘prosody’. In the administration of the MMART, task difficulty was progressed in difficulty across three domains, as follows:

1. Discrete units of expression (such as eyes only) progressed to combined units of expression (e.g., eyes and mouth).
2. Static stimuli (e.g., photo) progressed to dynamic (video); or single stimulus (vocal pitch) progressed to multiple stimuli (pitch and loudness).
3. Intensity of expression progressed from mild intensity to highly intense (face or voice).

### 2.3. Measures

Outcome measures were administered before the first session and after the last MMART session. We recorded number of enrolled participants completing the training program; number of sessions attended by each participant; and notation of active participation during treatment sessions. Participants were provided the opportunity after the treatment to provide their observation of any change in the following: their own feelings, their daily activities, interpersonal interactions, and outings outside the home. Responses were recorded.

Primary measure. The Florida Affect Battery (FAB) is a standardized measure of emotion recognition that assesses accuracy of recognition of both facial expressions and vocal expressions (prosody) of four emotions (happy, sad, fear, anger) and neutral; Table 1 summary is provided. Test-retest reliability in normal individuals ranged from 0.89 to 0.97 [21]. FAB domains #1–5 assess ability to accurately recognize facial expression of emotion. Domains #6–8 assess ability to recognize emotion content expressed in prosody. Domains #9 and #10 assess ability to simultaneously recognize combined affect and prosody. The possible score range for each domain is 0% to 100%.

Exploratory measures. The Attention Index of the Repeatable Battery for the Assessment of Neuropsychological Status (RBANS). Attention function is one of the critical cognitive processes required for identifying facial and prosody stimuli. Therefore, we administered the Attention Index of the RBANS [22], for which the scores are normed according to age, and which has shown good construct validity [23] and impairment sensitivity [23] for TBI, as well as sensitivity to identify impairment for those with moderate to severe TBI. For older adults, there was a report of four points as a minimal clinically important difference (MCID) in the Attention Index [24]. The RBANS Attention Index score is a standard score; for normal performance, the mean is 100, and one standard deviation is 15. Impairment is defined as a score that is below one standard deviation of the normal mean of 100 points. Therefore, in reporting our results for the RBANS attention index, we will identify an impairment as greater than one standard deviation below the mean of healthy adults, according to the published age norms for the RBANS Attention Index [22].

Emotion Recognition Test-short form (ERT). Speed of processing is important in encoding facial affect stimuli in everyday interpersonal interactions. Therefore, we assessed treatment response according to speed of processing for facial emotion recognition using the ERT, which is a speed test of facial emotion recognition that includes 48 assessment trials of male and female Caucasian faces (200 ms, timed stimuli) across differing emotions (anger, disgust, fear, happiness, sadness, surprise). Accuracy is reported as a standard score z-score; mean = 0; a score of −1 = 1 SD below the mean [25]. The ERT has been validated in neurological conditions [26,27,28].

Exploratory FAB subdomains. Descriptive data were generated for the FAB domains #9 and #10 because they assessed ability to simultaneously recognize combined facial affect and prosody information. That is, for these two domains, participants are required to perceive, process, and recognize the emotion content of both facial and prosody expression in order to complete any given task.

### 2.4. Statistical Analysis

For the primary measure, group pre-/post-treatment comparisons were made using a standard Wilcoxon signed-rank test for small sample size and IBM SPSSversion 30.0 software. The same procedure was used for the two secondary exploratory measures, the Attention Index and the ERT speed of processing test, as well as the exploratory analysis of FAB domains 9 and 10. For these latter, we interpreted a relaxed *p* value of less than 0.1 as a trend to be considered in this exploratory data for a pilot study. In the exploratory study for FAB domains #9 and #10, we generated descriptive statistics, and report the number of participants improving, declining, or maintaining the same score from pre-treatment to post-treatment.

In other exploratory analyses, we conducted a Pearson correlation for small sample sizes to identify potential correlations for future study; these included a correlation of domain #9 with domain #10, as well as correlations of potential contributing factors to the treatment response as measured by FAB domain #9 and domain #10. The potential contributing factors included the following: the PCL questionnaire, time since TBI, education level, BDI, and education level. For the ERT, two participants were lost to follow-up, due to ERT software version 0.1 problems. These two participants were dropped from the ERT analysis.

Descriptive data were also generated for the following: number of FAB domains improved or declined for each participant; pre-/post-data for individual participants for FAB domains 9 and 10; and number of enrolled participants completing the training program; number of sessions attended by each participant; notation of active participation during treatment sessions; and responses were organized in a table, regarding participants’ reports of any perceived change after treatment.

## 3. Results

### 3.1. Participants

We screened 17 Veterans, of which ten met inclusion criteria. Two of these Veterans declined prior to beginning the program, due to health and personal issues. Therefore, eight Veterans began and completed the MultiModal Affect Recognition Training (MMART) in eight sessions (4 weeks). Participants were screened, enrolled, and tested within 18 months. There were no adverse events. Participant characteristics are provided in Table 2.

All eight participants (100%) completed the MMART sessions (one participant missed one scheduled session and made it up). Of these eight participants, 100% (8/8) were able to actively participate in the content of all sessions (100% of sessions). Additionally, clinical notes provided evidence of active participation in each session for all participants. Participants reported some changes after treatment (Table 3).

### 3.2. Primary Measure

The Florida Affect Battery (FAB) showed a statistically significant pre-/post-treatment effect (Z (2.5); *p* = 0.012; effect size, 1.2). Pre- and post-treatment means were as follows: 89.4 (±5.7), 94.4 (±3.32), respectively.

### 3.3. Exploratory Measures

**Figure 1:**
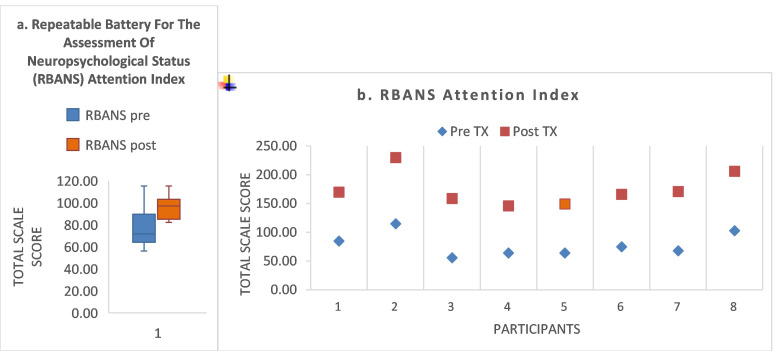
(**a**) For the RBANS measure, there was a significant treatment response (Z = 2.02, *p* = 0.04; effect size = 0.99). Descriptive statistics are shown in the boxplot as follows: Pre/post treatment means and SD, 78.7 (±20.8 SD; 95.9 ± 4.2 SD), respectively; with associated confidence intervals as follows: 61.1, 96.1; 86.0, 105.7, respectively. (Respective SEs: 7.5, 4.2). Pre/post treatment medians and interquartile range: 71.5 (±34.5) and 97.0 (±18), respectively. Pre-/post-treatment total range was as follows: 59, 33, respectively. Boxplot key: Colored box = interquartile range, black horizontal line = median, whisker plot = minimum and maximum. (**b**) For the RBANS measure, three subjects had normal scores at pre-treatment (S1, S2, S8) and maintained the normal score at post-treatment. Four participants exhibited baseline impairment (below one standard deviation of the mean threshold) and improved to within normal limits by post-treatment (S3, S5, S6, and S7). S4 was impaired at baseline and improved to within three points of the threshold for normal for his age group.

Attention. The RBANS attention index showed a significant pre-/post-treatment improvement (Z = 2.02, *p* = 0.04; effect size = 0.99). Pre- and post-treatment means were as follows: 78.8 (±20.8), 95.9 (±11.8), respectively. Full descriptive data are shown in Figure 1a and given in the figure legend.

Figure 1b (and Supplementary Table S1) shows that three subjects had normal scores at pre-treatment (S1, S2, S8) and maintained that score at post-treatment. Four participants exhibited baseline impairment (below one standard deviation of the mean threshold) and improved to within normal limits by post-treatment (S3, S5, S6, and S7). S4 was impaired at baseline and improved to within three points of the threshold for normal for his age group.

**Figure 2:**
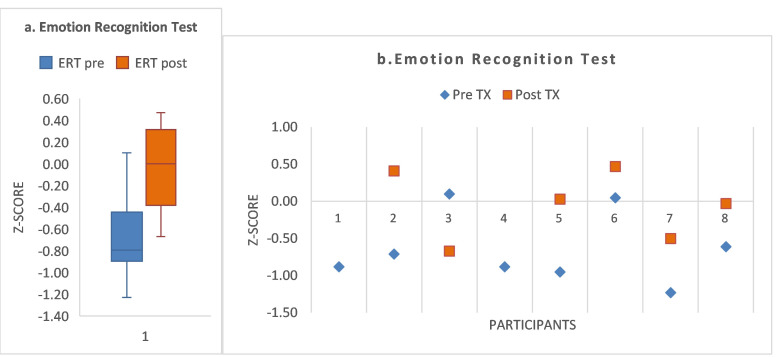
(**a**) For the ERT measure, the descriptive statistics are shown in the boxplot, as follows: Pre/post treatment means and SD, −0.56 (±0.54 SD; −0.05 ± 0.46 SD), respectively; with respective associated confidence intervals as follows: −1.1, +0.004; −0.54, +4.40. (Respective SEs: 0.22, 0.19). Pre/post treatment medians and interquartile range: −0.67 (±1.08) and 0.00 (±0.97), respectively. Pre-/post-treatment total range was as follows: 1.3, 1.14, respectively. Boxplot key: Colored box = interquartile range, black horizontal line = median, whisker plot = minimum and maximum. (**b**) For the ERT measure, there was a non-significant pre-/post-treatment result for the ERT as follows: Z = 1.36; *p* = 0.17, effect size, 0.75. Five of the participants exhibited a higher numerical score at post-treatment (S2, S5, S6, S7, S8); and two (S2 and S7) improved to more than one standard deviation above the mean (i.e., >one standard deviation above 1.0; S2 and S7; Supplementary Table S2).

Fast speed accurate identification of facial affect (Emotion Recognition Test (ERT)). There was a non-significant pre-/post-treatment result for the ERT as follows: Z = 1.36; *p* = 0.17, effect size = 0.75. Pre-/post-treatment z-score means increased as follows: 55 (±0.54); 0.05 (±0.46), respectively. Descriptive data are shown in Figure 2a and the figure legend. Additional individual subject descriptive data (Figure 2b and Supplementary Table S2) show that five of the participants exhibited a higher numerical score at post-treatment (S2, S5, S6, S7, S8); and two (S2 and S7) improved to more than one standard deviation above the mean (i.e., >one standard deviation above 1.0; S2 and S7; Supplementary Table S2).

**Figure 3:**
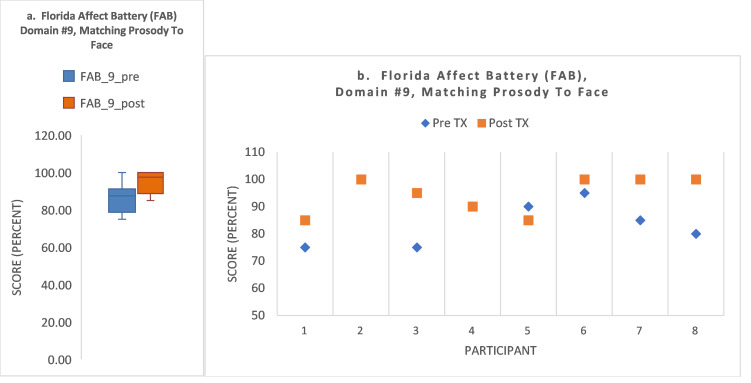
(**a**) For FAB domain #9, there was a trend toward significance (Z (1.9); *p* = 0.058). The descriptive statistics are shown in the boxplot as follows: pre/post treatment means and SD, 86.3 (±9.2 SD); and 94.4 (±6.8 SD); respectively; with associated 95% confidence intervals as follows: 78.6/93.9; 99.71/100, respectively. (Respective SEs: 3.2; 2.4.). Pre/post treatment medians (horizontal black line) and interquartile range (colored box): 87.5 (±17.5) and 97.5 (±13.8), respectively. Pre-/post-treatment total range was as follows: 25.0; 15.0, respectively (whisker plot, min and max scores). Boxplot key: Colored box = interquartile range, black horizontal line = median, whisker plot = minimum and maximum. (**b**) For FAB domain #9, 62.5% (5/8) of participants showed an improvement in post-treatment score. One (1/8) showed a ceiling effect, scoring 100% at both pre- and post-treatment (S2; pre-treatment blue diamond is obscured beneath the post-treatment red square). One (1/8) had no change throughout (S4; pre-treatment blue diamond is obscured beneath the post-treatment red square). One subject showed a decline in score (1/8; S5).

The FAB subdomain #9 task was to identify the spoken emotion content expressed and then select the correctly matching facial emotion expressed. There was a trend toward significance (Z (1.9); *p* = 0.058). Pre- and post-treatment group means were as follows, respectively: 86.3 (±9.2); 94.4 (±7.8). Full descriptive data are shown in Figure 3a and the figure legend.

Figure 3b shows that 5/8 subjects (62.5%) exhibited an increase in score (S1, S3, S6, S7, S8), shown by the higher values of the pink squares versus the blue diamond for each subject. Two had no change in score (S2, S4; 2/8; 25%), shown by the post-treatment pink square overlaying the blue pre-treatment dot; Figure 3b. One of these with no change had a pre-treatment ceiling effect of a perfect score (S2). One subject (1/8; 12.5%) showed a decline in score (S5).

**Figure 4:**
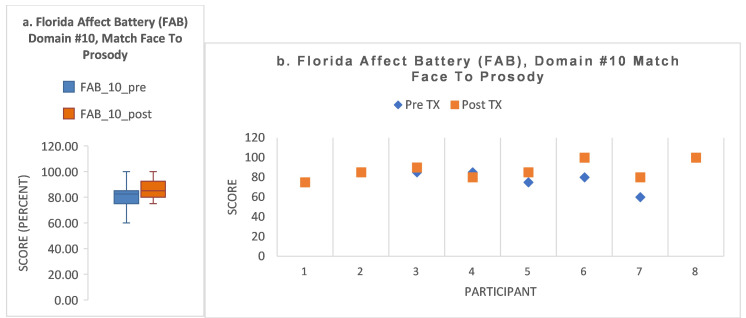
(**a**) For FAB domain #10, there was a non-significant result, trending toward significance (Z (1.63); *p* = 0.102). The descriptive statistics are shown in the boxplot as follows: Pre/post-treatment means and SD (80.6 (±11.5); 86.9 (±9.2)) with 95% confidence intervals as follows: 71.0/90.2 and 79.2/94.6, respectively. (Respective SEs: 4.1, 3.7). Pre/post-treatment medians and interquartile range: 82.5 (±10) and 85.0 (±17.5), respectively. Pre-/post-treatment total range was as follows: 40, 25, respectively. Boxplot key: colored box = interquartile range; black horizontal line = median; whisker plot = minimum and maximum. (**b**) For FAB domain #10, 50% (4/8) of participants showed an improvement in post-treatment score. There was no change for 25% (2/8) of participants (S1, S2; pre-treatment blue diamond obscured under post-treatment red square). One participant showed ceiling effect throughout (S8). One showed a decline (S4).

The FAB subdomain #10 task was to identify the facial emotion expressed and select the correctly matching audio sentence expressing that emotion. There was a trend toward significance (Z (1.63); *p* = 0.102). Pre- and post-treatment means were as follows, respectively: 80.6 (±11.5); 86.9 (±9.2). Full descriptive statistics are provided in Figure 4a. Figure 4b shows that four participants (4/8; 50%) had an increase in score (S3, S5, S6, S7). Three (3/8; 38%) had no change (S1, S2, S8). One participant (1/8; 12%) declined (S4).

Correlation analysis of domain #9 with domain #10 showed no significant association. For domain #9, there was no significant correlation to treatment response for any of the factors tested. For domain #10 treatment response, there was one factor with a trend toward significance, years post-TBI (r = 0.7; *p* = 0.052). Otherwise, there was only one other correlation of note, which was the correlation of the PCL-5 with the BDII (r = 0.94; *p* = <0.001).

### 3.4. Individual Differences

Because this is a pilot study with a small sample size, we are providing a short summary for participants. The information below is located in Table 4 and Supplementary Tables S1 and S2.

#### 3.4.1. Three Most Impaired Subjects at Baseline (Four to Seven FAB Subdomains Impaired (Table 4): S1, S5, S7)

- S5 had the most impaired FAB domains (seven impaired at baseline), with recovery to normal range for five of those domains by post-treatment. S5 showed baseline impairment in attention (score, 0.9th percentile), with a post-treatment increase in score, but only to the 16th percentile (Supplementary Table S1). According to accuracy of speed recognition of facial affect, S5 showed baseline impairment (−0.95) and recovery to within normal range at post-treatment (+0.03; Supplementary Table S2).
- S1 had five impaired FAB domains at baseline, with recovery of three FAB domains to normal range by post-treatment.
- For the Attention Index, S1 remained unchanged at the impaired level of the 16th percentile throughout.
- S7 had four impaired FAB domains at baseline, with recovery to normal range for three domains by post-treatment. S7 showed impaired baseline attention at the 1.6th percentile, improving by post-treatment to within the normal range (57th percentile). Speed of recognition accuracy improved from the impaired level (−1.23) to −0.5 by post-treatment.

Summarizing the above, we can note that they showed recovery of three to five FAB domains. One showed an improvement to normal on speed of accurate facial recognition and one showed an improvement to normal on the attention measure. The third had no change in the attention measure and no value was available for speed of accurate recognition. Domain #10 was one common remaining impaired FAB domain across all three subjects, which required simultaneous attention and judgement of facial affect and prosody.

#### 3.4.2. Subjects with Two Impaired FAB Domains at Baseline (S3, S4, S6)

- S3 showed baseline impairment in the two FAB domains requiring attention and encoding of both facial affect and prosody (domains #9, #10), improving to normal range by post-treatment. His baseline attention score was <1.0th percentile, but his attention score improved to normal range by post-treatment (57th percentile). In contrast to his gains in these measures, his ERT score declined from 0.1 to −0.67.
- S6 recovered to within normal range for domains #4 and #10. S6 showed a mixed response to treatment. Though he recovered the two impaired FAB domains (#4, #10) to within normal range, domains #2 and #8b1 showed a decline. His attention score improved 22 percentile points, impaired at the 5th percentile at baseline, improving to the 27th percentile by post-treatment. His speed of recognition improved from baseline (+0.05) to post-treatment (+0.47; a gain of 0.42 z-score points).
- These two participants, S3 and S6, who had only two impaired FAB domains at baseline, one of which was domain #10, recovered to normal range at baseline for domain #10. This finding is in contrast to those subjects who had a greater number of impaired FAB domains at baseline and did not improve to normal in domain #10 (previous subsection on S5, S1, S7).
- S4 was a ‘non-responder’ according to FAB domains #4 and #10. He showed impaired attention at baseline (<1st percentile), improving to only the 16th percentile by post-treatment.
- Summarizing the above, we can note that two subjects had recovery of 2/2 FAB domains, including domain #10. For these two subjects, one had a notable improvement in attention and the other had a notable improvement in speed of accurate recognition. A third subject was a non-responder, with two baseline-impaired FAB domains unchanged at post-treatment and no notable change in other measures.

#### 3.4.3. Subjects with Impairment in Only One FAB Domain at Baseline (S2, S8)

- S2 showed no post-treatment change in domain #10. Attention score was within normal range throughout. However, S2 had an improvement of z-score 1.12 points in speed of facial recognition (from pre-treatment −0.71 to post-treatment (+0.41)).
- S8 improved to within normal limits at post-treatment (domain #9). S8 showed an attention score within normal limits throughout. For speed of accurate facial recognition, S8 showed baseline impairment (−0.61) and improved to normal range by post-treatment (−0.03; z-score gain of 0.58).

Summarizing the above, we can note that one subject had no change in impaired domain #10, but the other subject improved to normal range for domain #9 at post-treatment. Both showed no impaired attention throughout. Both showed a notable improvement in speed of accurate facial recognition of emotion by post-treatment.

## 4. Discussion

This study contributes to the field in three important ways. First, on behalf of those with mTBI/PTSD, we developed and successfully administered a treatment protocol that targets recognition of emotion expression function. The current study is in contrast with existing studies that included only moderate/severe TBI and also did not address the comorbid condition of mTBI/PTSD. The results support the feasibility of providing the MMART protocol to veterans. Furthermore, and to our knowledge, this is the first study targeting impairment in recognition of emotion in both facial affect and prosody expression for those with mTBI/PTSD. Second, our pilot data, overall, showed statistically significant improvement in response to treatment for those with mTBI/PTSD, targeting impaired emotion recognition. Third, we gained insight into variation in treatment response across participants that can inform design of research studies, as well as precision (customized) clinical treatment plans.

### 4.1. Successful Administration of a New Multimodal Treatment Protocol, the MMART, That Targets Deficits in Emotion Recognition in Those with mTBI/PTSD

The MMART proved feasible for those with mTBI/PTSD according to a number of factors. There was 100% attendance in treatment sessions for all but one participant, who made up the missed session. This is consistent with other studies that enrolled moderate to severe TBI, with dropouts ranging from one to five participants [3,15,29].

In the current study, there was active participation noted for each treatment session attended, which reflects the successful reception of the training. Participant feedback indicated that treatment was meaningful to each individual. Emotion training could be a difficult topic for participants; indeed, in one other reported study, a participant withdrew because he ‘didn’t like talking about his emotions’ [16]. In our study, we constructed and delivered the MMART in a manner designed to be non-threatening and meaningful. Our success in that regard is supported by the very positive evidence provided by participants (Table 3).

According to this feedback from participants, there was generalization from the training content in at least two ways. First, for example, in the current study, we focused on recognition of emotion expression in others, and at the same time, participant comments showed an emerging improved sense of emotion in oneself. Second, although training consisted of working in a laboratory setting with a therapist, participants reported generalizing this training to positive changes in relationships within the family structure and in a work environment. The importance of generalization, through repetition and mastery of skills has been noted in Clinical Guidelines of Cognitive Rehabilitation and by several experts in the field of cognitive rehabilitation [30,31,32]. For those with moderate/severe TBI, others have reported improvements in participants’ ability to describe and differentiate emotions and emotional self-awareness in response to a treatment including psychoeducational lessons [16].

### 4.2. Statistically and Clinically Significant Improvement, for Those with Mild TBI/PTSD, in Primary and Secondary Measures in Response to Treatment Targeting Impaired Emotion Recognition

In the current study for those with mTBI/PTSD, there was a statistically significant treatment response according to the primary measure, the FAB, which assesses the gain in emotion recognition for happy, sad, fear, and anger. These encouraging current results for mTBI/PTSD are consistent with the few existing important prior studies of moderate/severe TBI [17]. Of note, these prior studies for severe/moderate TBI enrolled small samples and used only static pictures, such as the DANVA-2 or Ekman photos as the primary outcome measures. They reported improvement in recognizing emotions [33,34], with two studies reporting significant improvements [15,16].

Our current results showed a statistically significant gain in attention. In the MMART, attention training is not specifically targeted with conventional or general attention training, but participants are guided to focus attention on facial features and differences across any given feature for different emotion expression. In the same manner, participants are guided to focus attention on each element of prosody and differentiate between differences among emotions across any given prosody element. This type of practice may have improved performance on the RBANS Attention Index.

Our results are clinically important. For example, Table 4 provides an accounting of the number of baseline-impaired FAB domains that improved to within normal limits. The results are of clinical importance because the initial impairment improved to within normal limits for each of the domains listed for each participant (Table 4, column C). Additionally, results of clinical importance are supported by the participants’ anecdotal feedback on the effects of the training, such as their own newly emerging self-awareness, awareness of others’ emotion expression, improved personal relationships, and improvement in other interpersonal relationships.

Three MMART essential design features may explain, in part, these encouraging current findings. These three training features are inclusion of prosody; dynamic facial expressions; and discrete negative and positive emotion training. The first essential feature was that training specifically included identification of and discrimination between emotions of happy, sad, fear, and anger according to the prosody factors of vocal pitch, volume, and intensity. We should note that the MMART protocol offers training of both facial or prosody stimuli separately in the early sessions, followed by training of combined processing of both facial and prosody stimuli in sessions 7 and 8. In this study, there were two FAB domains (domains #9 and #10) that measured identification and matching of emotion expression in both facial and prosody stimuli in the same task items. Two participants improved in domain #9 (S1, S8; Table 4), but for domain #10, five participants did not improve (Table 4). One prior study for those with severe TBI [35] treated emotion prosody and showed mixed results. In a separate meta-analysis report, Murphy et al. [6] called attention to the lack of prosody training. They reported that the largest effect size of baseline emotion deficit in moderate/severe TBI occurred when there were concomitant stimuli of face, voice and body gestures.

The second essential feature of the MMART was the use of dynamic stimuli to train recognition of facial affect in others. The treatment in prior studies used only static images of facial affect for training that are not as ecologically valid as dynamic displays of emotion [3,16]. This type of limitation was cited in a review of emotion recognition training in severe TBI [17]. In contrast, the MMART was designed to utilize video stimuli during training of affect recognition. The dynamic video stimuli more closely replicate real-life situations, potentially resulting in greater learning.

A third essential design feature of the MMART was the important practice in recognition of discrete emotions [6], rather than the global emotion expression training of earlier studies [36]. Finally, the MMART included training in both positive and negative emotion recognition, which is important given the difference in difficulty for the two categories [6,37].

The MMART, equipped with these special features, could be uniquely effective and valuable in addressing the obstacles in treatment of recognizing emotion expression in others. Though there are no prior existing baseline data for mild TBI with regard to emotion function impairment, there is baseline information for moderate/severe TBI regarding severity of impairment in recognizing emotion expression in others; in fact, there was an important meta-analysis that described significantly poorer performance in emotion recognition for moderate/severe TBI versus healthy adults for the emotions of anger, disgust, and happiness, with potential problems for the emotions of fear, sadness, and surprise; there was a reported potential moderating variable of intensity and a recommendation was made for further study to obtain a clearer finding [6]. Of interest, that meta-analysis of 15 studies reported no difference in dysfunction of emotion recognition across TBI symptom severity level for moderate/severe individuals with TBI [6]. The need for an effective treatment such as the MMART for mTBI is supported not only by this published information for the impairment in those with moderate/severe TBI but also by our impaired baseline information for a sample of those with mTBI/PTSD, as well as their response to the MMART.

### 4.3. Individual Differences and Precision Neurorehabilitation (Customized Care)

The current study, for those with mTBI/PTSD, demonstrates that there were individual differences in treatment response for the FAB domains (Table 4), attention (Supplementary Table S1), speed of processing (Supplementary Table S2), and everyday function (Table 3). Our findings for mild TBI/PTSD are consistent with prior studies for moderate/severe TBI. In those prior studies, examples of potential predictors of individual treatment response of emotion recognition include the following: cognitive level, motivation, moderate/severe TBI symptom type and severity [3]; higher-function and age [15]. Future work could consider prior and/or current psychotherapy. The current work was focused on training the accurate identification of emotion expression in others through visual and auditory stimuli of face and voice. In future treatment development, a broader protocol could be considered, for example, with materials made available by the HUMAINE Database, which provides some naturalistic clips and labelling techniques [38].

Precision medicine is particularly relevant in neurorehabilitation [39], and especially in training of cognition and emotion function [32,40], and is relevant to this study on behalf of those with TBI [17]. Our results regarding individual differences support this contention as well.

In the current study, we employed a precision rehabilitation approach in a number of ways. First, we trained and progressed on four major factors, as follows: (a) discrete units of expression (such as eyes only) progressed to combined units of expression (e.g., eyes and mouth); (b) static stimuli (e.g., photo) progressed to dynamic (video); (c) single stimulus (vocal pitch) progressed to multiple stimuli (pitch and loudness); and (d) intensity of expression progressed from mild intensity to highly intense (face or voice). Second, progression was not conducted according to a strict time schedule; rather, progression was conducted according to the individual learning rate of each participant. Third, a calm, non-judgmental learning environment was ensured. Fourth, errors were identified in a productive manner and encouragement was provided. Fifth, at the outset, a strong working relationship was established between clinician and participant, with personal [41] difficulties and potential goals noted with regard to emotion recognition function; this process contributed to a motivating learning context. One prior study of moderate/severe TBI participants provided such a learning context by including psychoeducational lessons [42]; that study reported improvement in emotion self-awareness and ability to describe and differentiate emotions. Sixth, an overarching goal of the intervention was to empower the participant. To that end, individualized cues were provided, based on the careful observations of the clinician, in order to support the participant’s learning and independence in understanding his own learning process. Empowering the individual in such a way has been identified by neurorehabilitation specialists as an essential ingredient in successful neurorehabilitation [39,40,41,43].

### 4.4. The Florida Affect Battery Performance

The FAB was sensitive in a number of ways to impaired baseline performance and response to treatment. For example, the FAB identified three participants performing below normal for domain #8a and achieving gains by post-treatment within normal range. Second, the FAB also identified three participants performing below normal for domain #9 and recovering function at post-treatment to within normal range. Third, the FAB identified 6/8 participants with baseline impairment in domain #10, which required simultaneous attention and encoding of facial affect and prosody. This domain #10 did not improve for four subjects (three had no change, and one declined). One possible expansion of the current work is to test for factors possibly having an effect on treatment response, such as some of those factors included in the current work, as well as vocabulary, personality characteristics, and other relevant factors identified by others [44]. Such tests could include the following: Situational Test of Emotional Understanding and the Situation Test of Emotion Management, both published in 2008 [44], as well as the Geneva Multimodal Emotion Portrayals Core Set, which includes the addition of body action elements [45,46].

### 4.5. Study Limitations

The study limitations are consistent with those inherent in studies of small sample size, including limits of generalizability and caution required in interpreting results. In future studies, it would be important to conduct a larger, randomized controlled trial to evaluate efficacy. In addition, the results of study enrollment included only male participants, despite our broad catchment area geographically. Also, the age range was 31 to 53, according to the ‘convenience sample’ enrolled in order of application and qualification. These two limitations limit generalization to females and to other age groups, respectively. Emotion recognition is complex, and the complete array of complexity was not addressed in the current study. For example, in future work, there is a need for new interventions to train discrimination of intensity of emotion for those with TBI [37].

## 5. Conclusions/Impact

The MMART was feasibly administered for the sample of those with mTBI/PTSD. Treatment response was statistically significant and clinically important. The structure, content, and flexibility of the MMART allowed an individualized approach within sessions, possibly explaining some of the improved function measured. Along with their gains in emotion recognition, some individuals showed concomitant gains in attention function and/or speed of facial emotion recognition, indicating the potential benefit for additional sessions with content that would be more focused on attention training and speed of recognition. There were promising gains in the primary measure of emotion recognition, but at the same time, not all domains showed recovery to normal and not all subjects recovered to normal. These findings suggest potential value in increasing the number of training sessions. Subjects reported new insights and changes in everyday interpersonal relationships, suggesting potential value in expanding the intervention to include more specific targeting of emotion function within interpersonal relationships. Given that this is, to our knowledge, the first publication focusing on mTBI/PTSD and training targeted to recognition of emotion, there is an important clinical impact of these findings. The MMART is manualized and can be easily deployed to clinical practice. The development and testing of the MMART in this study was timely because both clinicians and research scientists have expressed the need for research evidence upon which to base clinical practice in the care of those with mTBI/PTSD [47,48,49]. For example, Hardy [50] states the following: “The fact that symptoms from each may be often indistinguishable suggests that assessment and treatment of mTBI and PTSD benefit from better clinical integration”.

## Figures and Tables

**Table 1:** Description of each domain of the Florida Affect Battery (FAB).

Domain (Number of Items)	Domain Description
	Affect (facial emotion recognition tasks)
1 (20)	Identify whether two faces with neutral emotions are the same or different people.
2 (20)	Identify whether two different people are facially expressing the same or different emotion.
3 (20)	Identify whether the facial expression of a face is ‘happy’, ‘sad’, ‘angry’, ‘frightened’, or ‘neutral’.
4 (20)	From five faces expressing different emotions, identify which one is expressing the target emotion given to you.
5 (20)	Identify the facial emotion on the left facial photo, then select the corresponding facial emotion from among five photos on the right side of the screen.
	Prosody (verbal/vocal emotion recognition tasks)
6 (16)	Listen to two different spoken sentences and identify whether the two sentences are the same (i.e., both a question or both a statement) or different (one is a question and one is a statement).
7 (20)	Listen to two different spoken sentences and identify if they are both expressing the same or different emotions.
8a (20)	For a given spoken sentence, identify the emotion expressed by the speaker (happy, sad, frightened, angry, or neutral).
8b	For a given spoken sentence, listen to how the sentence is said, not what is said. Identify the emotion expressed by the speaker (happy, sad, frightened, angry, or neutral).
8b1 ** (12)	Congruent items match emotion tone with content.
8b2 ** (24)	Incongruent items do not match emotion tone with content.
	Combined Affect and Prosody (facial and verbal/vocal combined emotion recognition tasks)
9	Listen to a sentence spoken in an emotional tone of voice and then select the facial expression (from three facial photos) that corresponds to the spoken sentence.
10	Inspect a photo showing a facial expression of emotion, then select from among three different spoken sentences, the one sentence that expresses the same emotion as the face.

** We separated FAB domain #8b into congruent or incongruent tasks (#8b1, #8b2). The possible range of scores for each domain is 0% to 100%.

**Table 2:** Participant Characteristics.

Characteristics	Age	Race	Vocational Status	Years of Education	Number of mTBI	Years from Last mTBI	PCL-5 Total Score	BDI-II Total Score
S1	50	African/ American	Unemployed	Some College	8	7	60	45 Severe
S2	50	African/ American	Volunteer 4 days/month	Master’s Degree	3	9	66	38 Severe
S3	48	African/ American	Unemployed	Some College	3	9	54	28 Moderate
S4	44	African/ American	Unemployed	High School	3	12	47	35 Severe
S5	40	African/ American	Unemployed	Some College	3	11	79	53 Severe
S6	38	Caucasion	Full-time	Master’s Degree	2	13	26	16 Mild
S7	53	Caucasion	Unemployed	Bachelor’s degree	1	16	28	16 Mild
S8	31	Caucasion	Part-time	Some College	2	10	57	37 Severe

Key: S = Subject; mTBI = mild traumatic brain injury; PCL-5 = Post-traumatic Brain Injury Symptom Checklist Version-5; BDI-II = Beck’s Depression Inventory Version-II; a total score of 0–13 indicates minimal depression, 14–19 mild depression, 20–28 moderate depression and 29–63 severe depression. Note: PCL-5 score of ≥33 indicates symptoms indicative of probable PTSD diagnosis.

**Table 3:** Participant Report.

Participant Number	Participant Report
1	“I think this is helping. I was taught from the age of 19 to kill a man, so this is a big departure from what I have done for many years. I only have given my family the angry facial expression. Now I try to use the happy facial and voice expression and my son hugged me out the blue this weekend. He hasn’t done that since my last deployment.”
2	“The role you play in society changes your behavior. I have become more involved in my church leadership and find that I need to have a more approachable demeaner. This is helping me become more aware of that.”
3	“I have the most difficulty expressing fear because we are taught as a soldier to get angry and aggressive when we feel fear. After training I have started to look at peoples’ emotions more carefully. I used to just look for their angry expression in order to avoid them.”
4	“I still have trouble controlling my emotions when I get mad, but now I am more aware of my expressions when I do get mad.”
5	“…confused about the emotions I am feeling after training. Now I notice the emotions I am expressing but don’t know how they got there. Have started to ask myself what I should be feeling.”
6	“…have become aware that my speech is more halted when I am out in public, even with people I know.”
7	“I feel like I have always been good at distinguishing facial expressions but now I am more aware of the tone of voice people are expressing. I am more aware of how I am expressing myself to others with both my facial and voice expressions. My wife says that I rarely shared my emotions before and now I state how I am feeling. She said my facial expressions were flat and now I seem more aware and use a greater variety of facial expressions.”
8	“I have been having difficulty with relationships with family and friends. Now I step back and try to process what they are expressing.”

Key for Table 3: “…”, these quotation marks indicate that the above information is direct quotes either written by the participant or verbally expressed and noted verbatim by the clinician.

**Table 4:** Individual Differences for Florida Affect Battery (FAB): Number of Domains Improved, Declined, or No-Change, by Subject.

A. Subject	B. Number of Domains Impaired at Baseline (Domain Number #)	C. Number of “Baseline-Impaired”		D. Number of ‘Normal Baseline’ Domains, Declined (Domain Number #)
		Domains Improved (Domain Number #)	Domains, No Change (Domain Number #)	
S1	5 (#’s 5, 6, 8a, 9, 10)	3 (#’s 5, 8a, 9)	2 #’s (6, 10)	1 (#3)
S2	1 (#10)	0	1 (# 10)	0
S3	2 (#’s 9, 10)	2 (#’s 9, 10)	0	0
S4	2 (#’s 4, 10)	0	2 (#’s 4, 10)	0
S5	7 (#s 1, 3, 4, 5, 6, 8a,10)	5 (#s 1, 3, 5, 6, 8a)	2 (#’s 4, 10)	0
S6	2 (#’s 4, 10)	2 (#’s 4, 10)	0	2 (#’s 2, 8b1)
S7	4 (#’s 7, 8a, 8b1, 10)	3 (#’s 7, 8a, 8b1)	1 (#10)	1 (#5)
S8	1 (# 9)	1 (# 9)	0	0

Key: Domains # 1–5 measured recognition of facial affect. Domains # 6–8b2 measured recognition of prosody. Domains # 9 and 10 measured recognition of facial affect and prosody.

## Data Availability

The original contributions presented in this study are included in the article/Supplemantary Materials. Further inquiries can be directed to the corresponding author.

## Supplementary Materials

The following supporting information can be downloaded at: https://www.mdpi.com/article/10.3390/brainsci15070728/s1, Supplementary Table S1: RBANS Attention Index; Table S2: Emotion Recognition Test (ERT).
