# Correction: Waid-Ebbs et al. Response to Training in Emotion Recognition Function for Mild TBI/PTSD Survivors: Pilot Study. *Brain Sci.* 2025, *15*, 728

**DOI:** 10.3390/brainsci15101063

**Published:** 2025-09-30

**Authors:** J. Kay Waid-Ebbs, Kristen Lewandowski, Yi Zhang, Samantha Graham, Janis J. Daly

**Affiliations:** 1 Department of Veterans Affairs (VA), Rehabilitation Research and Development, Brain Rehabilitation Research Center, Gainesville, FL 32608, USA; yi.zhang@va.gov (Y.Z.); samantha.graham2@va.gov (S.G.); jjd17@case.edu (J.J.D.); 2 Department of Speech, Language, and Hearing Sciences, College of Public Health and Health Professions, University of Florida, Gainesville, FL 32611, USA; kristen.lewandowski@va.gov; 3 Department of Physical Therapy, College of Public Health and Health Professions, University of Florida, Gainesville, FL 32611, USA; 4 Department of Neurology, School of Medicine, Case Western Reserve University, Cleveland, OH 44106, USA

## Missing Acknowledgments

In the original publication [1], there was a missing sentence in the Acknowledgments Section.

The correct missing sentence appears below:

**Acknowledgments**: Susan A. Leon, PhD, CCC-SLP, developed and manualized a large portion of the treatment protocol used in this study.

The scientific conclusions are unaffected.
