# Supplementary material for: Response to Training in Emotion Recognition Function for Mild TBI/PTSD Survivors: Pilot Study
Source: Brain Sci. 2025 Jul 8;15(7):728. doi: 10.3390/brainsci15070728 (PMC12293806; doi:10.3390/brainsci15070728)
Supplement: Supplementary file 1 [file brainsci-15-00728-s001.zip › brainsci-3694291-supplementary.pdf]

**Supplementary Table S1. RBANS Attention Index**			
	Pre-treatment Index Score (Percentile)	Post-treatment Index Score (Percentile)	Difference
Subject ID			
S1	85 (16%)	85 (16%)	(0)
S2	115 (84%)	115 (84%)	(0)
S3	56 (.01%)	103 (57%)	(47)
S4	64 (0.9%)	82 (11%)	(18)
S5	64 (0.9%)	85 (16%)	(21)
S6	75 (5%)	91 (27%)	(16)
S7	68 (1.6%)	103 (57%)	(35)
S8	103 (57%)	103 (57%)	(0)

**Supplementary Table S2. Emotion Recognition Test (ERT)**			
	Pre-treatment (z-score scale used by the ERT)	Post-treatment (z-score scale used by the ERT)	Mean Difference (z-score scale used by the ERT)
Subject ID			
1	-0.88	.	.
2	-0.71	0.41	**1.12**
3	0.1	-0.67	-0.77
4	-0.88	.	.
5	-0.95	0.03	**0.98**
6	0.05	0.47	**0.42**
7	-1.23	-0.5	**0.73**
8	-0.61	-0.03	**0.58**
